# Sensory phenotypes in complex regional pain syndrome and chronic low back pain—indication of common underlying pathomechanisms

**DOI:** 10.1097/PR9.0000000000001110

**Published:** 2023-11-15

**Authors:** Iara De Schoenmacker, Laura Sirucek, Paulina S. Scheuren, Robin Lütolf, Lindsay M. Gorrell, Florian Brunner, Armin Curt, Jan Rosner, Petra Schweinhardt, Michèle Hubli

**Affiliations:** a Spinal Cord Injury Center, Balgrist University Hospital, University of Zurich, Zurich, Switzerland; b Neuroscience Center Zurich, University of Zurich, Zurich, Switzerland; c Department of Chiropractic Medicine, Integrative Spinal Research Group, Balgrist University Hospital, University of Zurich, Zurich, Switzerland; d Department of Neurology, University Hospital Bern, Inselspital, University of Bern, Bern, Switzerland; e Physical Medicine and Rheumatology, Balgrist University Hospital, Zurich, Switzerland; f Danish Pain Research Center, Department of Clinical Medicine, Aarhus University, Aarhus, Denmark; g Alan Edward Center for Research on Pain, McGill University, Montreal, QC, Canada

**Keywords:** Chronic pain, Sensory function, Pain phenotyping, Cluster analysis, Quantitative sensory testing

## Abstract

**Introduction:**

First-line pain treatment is unsatisfactory in more than 50% of chronic pain patients, likely because of the heterogeneity of mechanisms underlying pain chronification.

**Objectives:**

This cross-sectional study aimed to better understand pathomechanisms across different chronic pain cohorts, regardless of their diagnoses, by identifying distinct sensory phenotypes through a cluster analysis.

**Methods:**

We recruited 81 chronic pain patients and 63 age-matched and sex-matched healthy controls (HC). Two distinct chronic pain cohorts were recruited, ie, complex regional pain syndrome (N = 20) and low back pain (N = 61). Quantitative sensory testing (QST) was performed in the most painful body area to investigate somatosensory changes related to clinical pain. Furthermore, QST was conducted in a pain-free area to identify remote sensory alterations, indicating more widespread changes in somatosensory processing.

**Results:**

Two clusters were identified based on the QST measures in the painful area, which did not represent the 2 distinct pain diagnoses but contained patients from both cohorts. Cluster 1 showed increased pain sensitivities in the painful and control area, indicating central sensitization as a potential pathomechanism. Cluster 2 showed a similar sensory profile as HC in both tested areas. Hence, either QST was not sensitive enough and more objective measures are needed to detect sensitization within the nociceptive neuraxis or cluster 2 may not have pain primarily because of sensitization, but other factors such as psychosocial ones are involved.

**Conclusion:**

These findings support the notion of shared pathomechanisms irrespective of the pain diagnosis. Conversely, different mechanisms might contribute to the pain of patients with the same diagnosis.

## Abstract 2

Supplemental Digital Content is Available in the Text.

Two clusters of sensory phenotypes were identified across various chronic pain diagnoses, indicating common pathophysiological mechanisms irrespective of the pain diagnosis.

## 1. Introduction

Chronic pain poses a substantial burden for affected individuals, the healthcare system, and society at large. Despite advances in understanding the pathomechanisms of chronic pain, patient heterogeneity in terms of underlying mechanisms limits positive treatment outcomes. Identifying more homogenous subgroups of chronic pain patients with common pathomechanisms might enable more targeted treatment strategies.

To this end, the field of pain research has moved towards “phenotyping” strategies to infer underlying pathomechanisms of chronic pain.^15^ One approach to subgroup patients is stratification based on sensory phenotypes assessed by quantitative sensory testing (QST). Patients with chronic primary pain,^8,12,17,27,38^ chronic neuropathic pain,^3,37,43,45,54^ and chronic secondary musculoskeletal pain^18,39,41^ were subgrouped based on sensory phenotypes using cluster analysis approaches. For instance, Baron et al.^3^ identified 3 clusters in peripheral neuropathic pain patients using QST in the painful body area. These clusters were then compared with sensory phenotypes of clinical and experimental studies investigating sensory loss^4,20,55^ or peripheral and central sensitization^52^ in human surrogate models.^33,50^ This allowed for linking of the assessed sensory phenotypes to potential underlying pathomechanisms.

Other studies related sensory phenotypes to psychological factors and pain characteristics to provide further insights into potential underlying mechanisms.^8,18,24,41^ For example, in patients with osteoarthritis^18^ and neuropathic pain,^24^ increased anxiety and depression scores have been documented in subgroups with decreased thermal and mechanical pain thresholds. Regarding pain characteristics, most studies reported higher clinical pain intensities in patients with increased pain sensitivity as assessed by QST.^18,27,37,38^ Moreover, widespread hyperalgesia was reported in patients with extended but not local back pain^23^ and in patient subgroups with increased temporal summation of pain,^39,41^ a proxy for central sensitization.^1^

Although phenotyping based on sensory profiles has proven useful to detect patient subgroups with common pathomechanisms, previous studies were limited to similar chronic pain cohorts and mainly focused on the most painful area.^3,41^ For example, Baron et al.^3^ have demonstrated that patients with different etiologies of peripheral neuropathic pain, such as polyneuropathy, radiculopathy, peripheral nerve injury, and postherpetic neuralgia, may present with similar sensory phenotypes. Although the observation of common underlying mechanisms in peripheral neuropathic pain is clinically relevant, it remains to be determined whether these observations also apply to other types of pain that do not primarily involve a lesion or disease of the somatosensory system.

The aim of this study was to investigate the presence of common or dissimilar sensory phenotypes across different chronic pain cohorts (complex regional pain syndrome [CRPS] and chronic low back pain [LBP]) as indicators of conjoint or distinct potential underlying mechanisms, respectively. We hypothesized that within a population of different chronic pain patients, clusters based on sensory phenotypes will be formed irrespective of the pain diagnosis. Also, we hypothesized that patients with sensory phenotypes, including sensory/pain hypersensitivities, would present with more intense, widespread chronic pain and elevated depression, anxiety, and pain catastrophizing scores.

## 2. Methods

### 2.1. Study population

Chronic pain patients and age-matched and sex-matched healthy controls (HC) (aged between 18–80 years) were recruited between November 2019 and April 2022. Specifically, we included chronic pain patients with CRPS type I and nonspecific LBP. This chronic pain cohort selection was a convenience sample because all patients were recruited in collaboration with the Department of Rheumatology and Chiropractic Medicine at the Balgrist University Hospital in Zurich, Switzerland. More specifically, patients with CRPS were recruited from the Department of Rheumatology and had to fulfill the clinical Budapest Criteria^25^ at inclusion, which was examined by a rheumatologist (F.B.) with CRPS expertise. Patients with LBP were recruited through the Department of Chiropractic Medicine and through advertisements in Swiss Chiropractic practices and Chiropractic care journals. Exclusion criteria for patients (CRPS and LBP) were pain for less than 3 months; neurological (eg, polyneuropathy, radiculopathy), systemic (eg, autoimmune disease, diabetes), or psychiatric diseases; pregnancy; or inability to follow study instructions. In addition, LBP patients were excluded in case of primary pain complaints other than LBP or the presence of “red flags” (eg, signs of infection, fractures, inflammation).^48^ Healthy controls had the same exclusion criteria with the addition of not having acute pain, a history of chronic pain (>3 months), or LBP lasting longer than 3 consecutive days during the last year. Moreover, normal sensory integrity of all participants (patients and HC) was tested before experimental study procedures in a nonpainful and nonaffected area, ie, hand or shoulder, by bedside sensory testing (vibration, thermosensation, pinprick, and light touch). Written informed consent was obtained from each participant, and all experimental procedures were conducted in accordance with the Declaration of Helsinki. The study was approved by the local ethics board “Kantonale Ethikkommission Zürich, KEK” (EK-04/2006, PB_2016-02051 and PB_2019-00136, clinicaltrial.gov number: NCT02138344 and NCT04433299).

### 2.2. Study protocol

This study was part of a larger project called Clinical Research Priority Program (CRPP) Pain funded by the University of Zurich. The experimental protocol comprised a comprehensive testing battery (2 visits of 3 hours each), including evaluation of clinical pain characteristics, neurophysiological assessments, experimental pain paradigms, and psychological and pain questionnaires. The data presented in this article include (1) QST; (2) assessment of the pain characteristics; and (3) psychological questionnaires (pain catastrophizing scale [PCS]^51^ and hospital anxiety and depression scale [HADS]).^56^ Quantitative sensory testing and clinical pain characteristics were assessed during the first visit, whereas electronic versions of the questionnaires were completed separately from the 2 visits within 1 week of the first visit.

### 2.3. Assessment of clinical pain characteristics

The spatial pain extent was assessed using pain drawings, as described by Rosner et al.^47^ Only painful areas associated with the condition were considered for further analysis (syndrome-specific pain for CRPS patients and pain between the 12th rib and the gluteal fold and in the legs for LBP patients). Moreover, the current pain intensity of the most painful area was specified on a numeric rating scale (NRS) from 0 to 10 (0: no pain, 10: worst pain imaginable). The average and maximal pain intensity of the past 4 weeks and the self-reported evoked pain to temperature, light touch, and pressure were assessed through questionnaires (part of the painDETECT questionnaire^19^). Answers to the self-reported evoked pain questions were transformed into numbers between 0 and 5 for further analysis (0: never, 1: hardly noticed, 2: slightly, 3: moderately, 4: strongly, 5: very strongly). In addition, information about regular pain medication intake was obtained through questionnaires. Pain medication was classified according to the Anatomical Therapeutic Chemical/Defined Daily Dose classification by the World Health Organization (https://www.whocc.no/atc_ddd_index/) into categories of anti-inflammatory and antirheumatic products, analgesics (opioidergic and nonopioidergic), anticonvulsants, psycholeptics, and psychoanaleptics.

### 2.4. Quantitative sensory testing

The QST protocol was performed by trained experimenters and based on the guidelines of the German Research Network on Neuropathic Pain (DFNS).^46^ In every patient, the respective area with the typically highest pain intensity (most painful area) and a remote, pain-free body area (control area) were assessed. The control area was typically the nondominant hand or the hand contralateral to the most painful area in case of CRPS. Complex regional pain syndrome patients with an affected hand had the contralateral shoulder as control area because previous studies have shown that symptoms can spread to the contralateral limb.^44^ Individually, age-matched and sex-matched HC were measured in the same areas as the patients. The QST protocol performed in the most painful area consisted of the entire QST protocol. The main focus of performing QST in the control area was to assess widespread hyperalgesia and not sensory loss. Because of time constraints, we only performed QST measures assessing sensory gain of function in the control area (cold pain threshold [CPT], heat pain threshold [HPT], mechanical pain threshold [MPT], stimulus-response function [SR-function], pressure pain threshold [PPT], and windup ratio [WUR]). Regarding the SR function, only 2 (instead of 5) stimulations of each stimulus were applied in the control area. In accordance with the DFNS protocol, the control area was measured first. Quantitative sensory testing measures were z-transformed for each participant using the eQuiSTA software (version 1.3.4) provided by the DFNS.

### 2.5. Statistical analysis

Statistical analysis was performed in R Studio statistical software (R version 4.1.2 for Windows). An α-level of 0.05 was used for statistical inference, corrected for multiple comparisons by a Benjamini–Hochberg correction. Data were tested for normality by a Shapiro–Wilk test.

#### 2.5.1. Demographics, pain characteristics, and psychological factors

Age, height, and weight were compared between patients (pooled) and HC using an unpaired *t* test or Wilcoxon signed-rank test. A χ^2^ test was used to compare the sex proportion within patients and HC. Pain characteristics and self-reported evoked pain were compared between CRPS and LBP patients by a *t* test or Wilcoxon signed-rank test. In addition, analysis of variance or Kruskal–Wallis rank sum test was used to compare psychological questionnaire scores between the 3 cohorts (CRPS, LPB, and HC).

#### 2.5.2. Cluster analysis

The QST z-scores (excluding paradoxical heat sensation [PHS] and dynamic mechanical allodynia [DMA]) were averaged into 4 different QST domains to reduce the dimensionality for the cluster analysis: (1) thermal detection; (2) thermal pain; (3) mechanical detection; and (4) mechanical pain. The thermal detection domain incorporated the warm and cold detection threshold (WDT and CDT, respectively) and the thermal sensory limen (TSL). The thermal pain domain included the CPT and HPT. The mechanical detection domain comprised the mechanical and vibration detection threshold (MDT and VDT, respectively). Finally, the mechanical pain domain consisted of the MPT, mechanical pain sensitivity (MPS) (calculated from the SR function), PPT, and WUR. This approach of dimension reduction was chosen because the main goal was to focus on the loss or gain of function within the 2 tested modalities (thermal and mechanical). The minimum and maximum z-score for each QST domain was set to ±4 SD to minimize the possibility of cluster assignments due to single extreme outlier values. K-means clustering was applied to the 4 QST domains only including the data of the most painful area of all patients (without HC). The K-means clustering was performed by the NbClust() function of the NbClust package using Euclidean distance and k ranging from 2 to 10. The NbClust() function provides 30 indices determining the optimal number of clusters. The most frequently determined optimal cluster number is then chosen as final optimal number of clusters. To investigate whether the final result of optimal number of clusters was robust, the same cluster analysis was repeated using bootstrapped data with 1000 iterations. In addition, a hierarchical clustering approach within the same NbClust() function (“average” method) was performed. The optimal number of clusters and the percentage of same cluster allocations was specified between the K-means and hierarchical clustering method.

#### 2.5.3. Differences between patient clusters and healthy controls

The relative chronic pain cohort contribution per cluster was assessed. Differences between each patient cluster and HC regarding QST domain z-scores (each domain separately) in the painful and control area, pain characteristics, and psychological factors were tested by general linear models with cohort (CPRS, LBP, and HC) as covariate. The Tukey HSD test was applied following significant general linear models as a post hoc test. Age, PHS, and DMA were compared between the clusters and HC using a Kruskal–Wallis rank sum test. Differences between clusters in sex distribution and intake of pain medication (yes/no) were tested by χ^2^ tests. In addition, the proportion of patients presenting with significant loss or gain of function (>|1.96 SD|) per cluster and HC was calculated (based on the DFNS z-scores).

## 3. Results

### 3.1. Cohort-specific differences

A total of 81 patients and 63 age-matched and sex-matched HC were recruited. In particular, this included 20 patients with CRPS and 61 patients with LBP. Of the 144 study participants, 3 patients and 2 HC were excluded. In more detail, 1 patient (CRPS) did not tolerate the study protocol, 1 patient (LBP) and 2 HC showed evidence of a neurological condition during sensory bedside testing, and 1 patient (LBP) displayed signs of a psychiatric condition, which was identified after inclusion. There were no differences regarding sex distribution (patients: 68% female, HC: 56% female, *P* = 0.140), age (patients: 50 ± 16 y, HC: 48 ± 16 y, *P* = 0.629), height (patients: 170±8 cm, HC: 172 ± 8 cm, *P* = 0.163), or weight (patients: 70 ± 13 kg, HC: 70 ± 12 kg, *P* = 0.977) between patients and HC. Table 1 illustrates the pain characteristics of all patients. A detailed list of the most painful and pain-free control areas of the patients can be found in the supplementary Table S1, http://links.lww.com/PR9/A211. Table 2 illustrates the psychological questionnaire scores of patients and HC. Of 78 patients, 27 regularly took pain medication. The supplementary Table S2, http://links.lww.com/PR9/A211 illustrates the intake of pain medication in more detail.

**Table 1:** Pain characteristics and self-reported evoked pain of chronic pain patients.

	Pain characteristics				
	All patients	CRPS (N = 19)	LBP (N = 59)	W	*P*
Current pain intensity [NRS]	3.4 ± 2.2 (0–8)	4.6 ± 2.2 (1–8)	3.1 ± 2.0 (0–7)	357.5	**0.018**
Average pain intensity over 4 wk [NRS]	4.4 ± 2.0 (1–10)	5.5 ± 2.4 (1–10)	4.0 ± 1.7 (1–8)	331.0	**0.015**
Maximal pain intensity over 4 wk [NRS]	6.2 ± 2.2 (2–10)	7.4 ± 2.2 (2–10)	5.8 ± 2.1 (2–10)	323.0	**0.008**
Spatial pain extent [%]	2.9 ± 3.8 (0.1–19.5)	6.6 ± 5.7 (0.5–19.5)	1.7 ± 1.5 (0.1–7.6)	263.0	**0.001**
Pain duration [months]	113 ± 149 (4–670)	29 ± 22 (6–98)	141 ± 161 (4–670)	758.5	**0.016**

	Self-reported evoked pain (from the painDetect)				
	All patients	CRPS (N = 19)	LBP (N = 59)	W	*P*
Temperature [score]	1.4 ± 1.7 (0–5)	3.5 ± 1.5 (0–5)	0.7 ± 1.1 (0–4)	114.0	**<0.001**
Light touch [score]	0.8 ± 1.4 (0–5)	2.5 ± 1.8 (0–5)	0.2 ± 0.4 (0–2)	150.5	**<0.001**
Pressure [score]	2.5 ± 1.4 (0–5)	3.4 ± 1.1 (1–5)	2.2 ± 1.4 (0–5)	299.0	**0.004**

Data are presented as mean ± standard deviation (range). The *P* value indicates significant differences between the 2 chronic pain cohorts. Significant results are printed bold.

CRPS, complex regional pain syndrome; LBP, low back pain; NRS, numeric rating scale.

**Table 2:** Psychological factors of chronic pain patients and healthy controls.

	CRPS	LBP	HC	X^2^	*P*
HADS [score]	15 ± 8 (0–26)*†	9 ± 6 (0–22)*‡	5 ± 4 (0–21)†‡	28.4	**<0.001**
PCS [score]	22 ± 12 (0–42)*†	13 ± 10 (0–36)*‡	5 ± 7 (0–23)†‡	38.7	**<0.001**

Data are presented as mean ± standard deviation (range). Significant results are printed bold.

* Post hoc analysis significance: CRPS-LBP.

† Post hoc analysis significance: HC-CRPS.

‡ Post hoc analysis significance: HC-LBP.

CRPS, complex regional pain syndrome; HADS, hospital anxiety and depression scale; HC, healthy controls; LBP, low back pain; PCS, pain catastrophizing scale.

### 3.2. Cluster differences in sensory phenotypes and demographics

**Figure 1.**
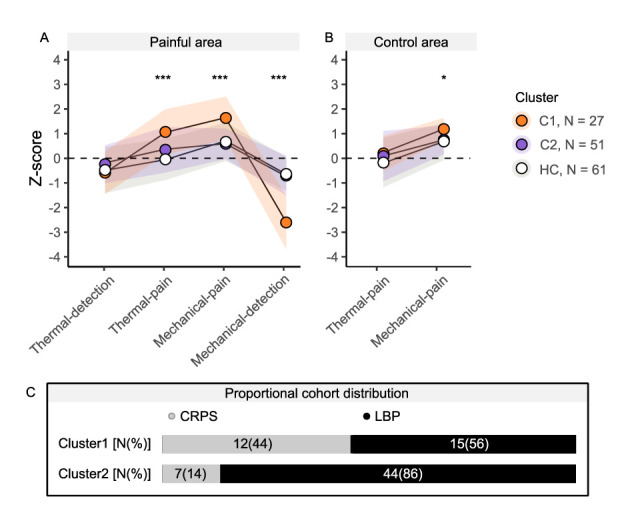
Sensory profiles and proportional cohort distribution of the 2 patient clusters. (A) Sensory profiles measured in the painful area of the 2 clusters and healthy controls (HC). (B) Sensory profiles measured in the control area of the 2 clusters and HC. Illustrated are the averaged z-scores for each QST measurement domain (thermal detection, thermal pain, mechanical pain, mechanical detection). (C) Relative cohort distributions for each cluster. *P < 0.05, **P < 0.01, ***P < 0.001. CRPS, complex regional pain syndrome; LBP, low back pain; QST, quantitative sensory testing.

K-means clustering revealed N = 2 as the optimal number of clusters (for quality criteria, see supplementary Table S3, http://links.lww.com/PR9/A211). The stability of the optimal number of clusters was confirmed by (1) the 1000 iterations based on the bootstrapped data (see supplementary Figure S1, http://links.lww.com/PR9/A211) and (2) the hierarchical clustering method. Hierarchical clustering also provided 2 clusters as the optimal number of clusters with a 91% agreement in cluster allocation. The sensory profiles of the 2 clusters based on QST measurements within the patients' most painful area are displayed in Figure 1A. Besides the thermal detection domain, there were significant differences between the patient clusters and HC for the remaining QST domains (thermal detection: F(2,135) = 1.91, *P* = 0.182; mechanical detection: F(2,135) = 62.32, *P* < 0.001; thermal pain: F(2,135) = 13.62, *P* < 0.001; mechanical pain: F(2,135) = 8.56, *P* < 0.001). Cluster 1 (N = 27) presented with significant gain of function compared with HC in the thermal (*P* = 0.002) and mechanical (*P* < 0.001) pain domains and significant loss of function in the mechanical detection domain (*P* < 0.001). Cluster 2 (N = 51) did not show differences compared with HC in any of the QST domains (mechanical detection: *P* = 0.915; thermal pain: *P* = 0.063; mechanical pain: *P* = 0.780). There was no difference in the presence of PHS or DMA across the clusters and HC (DMA: X^2^ = 3.61, *P* = 0.164; PHS: X^2^ = 3.23, *P* = 0.199). Within the control area, the mechanical pain domain was different between the 2 clusters and HC (F(2,133) = 5.093, *P* = 0.011, Figure 1B). Here, cluster 1 showed more mechanical gain of function compared with HC (*P* = 0.019), which was not observed when comparing cluster 2 with HC (*P* = 0.860). There was no difference in the thermal pain domain in the control area between cluster 1, cluster 2, and HC (F(2,133) = 1.60, *P* = 0.206).

The 2 chronic pain cohorts were distributed across both clusters with different relative proportions (Fig. 1C). The relative sex distribution was not different between cluster 1, cluster 2, and HC (X^2^ = 5.841, *P* = 0.067, female proportion: C1 = 22/27 (81%), C2 = 31/51 (61%), HC = 34/61 (56%)). In addition, age was not different between cluster 1, cluster 2, and HC (X^2^ = 0.881, *P* = 0.644, age (mean ± SD): C1 = 47.7 ± 15.5 y, C2 = 50.7 ± 15.9 y, HC = 48.4 ± 16.0 y). Moreover, there was no difference in regular intake of pain medication between the clusters (X^2^ = 0.685, *P* = 0.408). Here, 11 of 27 patients (41%) of cluster 1 and 16 of 51 patients (31%) of cluster 2 regularly took pain medication.

**Figure 2.**
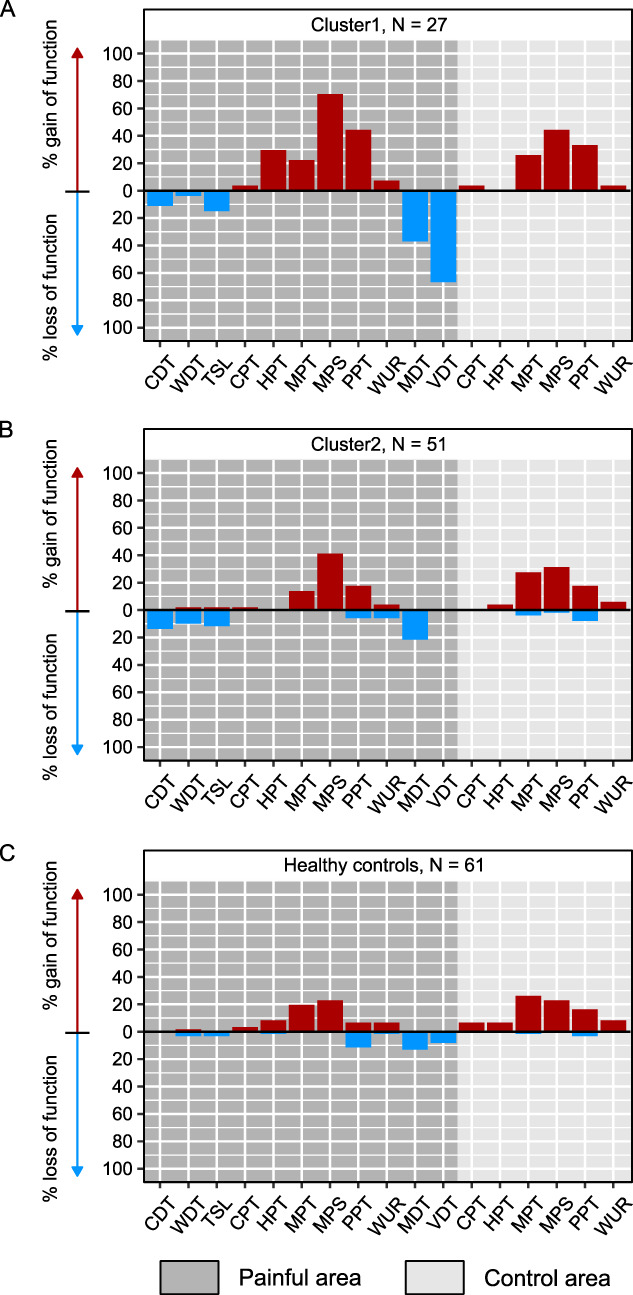
Relative number of participants with significant loss (blue) or gain (red) of function (based on the DFNS z-scores) for each cluster and healthy controls. (A) Cluster 1, (B) cluster 2, and (C) healthy controls are illustrated separately. CDT, cold detection threshold; CPT: cold pain threshold; DFNS, German research network on neuropathic pain; HPT, heat pain threshold; MDT, mechanical detection threshold; MPS, mechanical pain sensitivity; MPT, mechanical pain threshold; PPT, pressure pain threshold; TSL, thermal sensory limen; VDT, vibration detection threshold; WDT, warm detection threshold; WUR, wind-up ratio.

Figure 2 illustrates the relative number of patients per cluster or of HC with a significant loss/gain of function for each QST measure within the most painful and control area. In the most painful area, up to ∼70% of cluster 1 (Fig. 2A) presented with a combination of mechanical loss (detection threshold) and gain of function (pain threshold). Cluster 2 (Fig. 2B) and HC (Fig. 2C) showed a similar pattern to cluster 1, with a combination of mechanical loss and gain of function, however, to a lower degree (∼20–40%). In the control area, a gain of function, especially for mechanical pain thresholds, was observed in both clusters and HC, which was most frequently seen in cluster 1 (∼45%) and least frequently in HC (∼25%).

### 3.3. Cluster differences in pain characteristic and psychological factors

The differences in pain characteristics between the 2 clusters are illustrated in Table 3. Interestingly, cluster 1 reported higher maximal pain intensities and larger spatial pain extents compared with cluster 2. Moreover, cluster 1 more frequently had temperature, light touch, and pressure evoked pain compared with cluster 2.

**Table 3:** Analysis of pain characteristics (between the clusters) and psychological factors (between clusters and healthy controls).

	Pain characteristics			
	Cluster 1	Cluster 2	F	*P*
Current pain intensity	3.6 ± 2.6 (0–8)	3.4 ± 1.9 (0–7)	0.14	0.782
Average pain intensity over 4 wk	5.0 ± 2.2 (1–10)	4.0 ± 1.9 (1–8)	3.98	0.062
Maximal pain intensity over 4 wk	7.2 ± 1.8 (3–10)	5.7 ± 2.3 (2–10)	9.43	**0.006**
Spatial pain extent	4.1 ± 5.1 (0.1–19.5)	2.3 ± 2.7 (0.1–14.2)	5.99	**0.024**
Pain duration	118 ± 137 (6–453)	111 ± 157 (4–670)	0.05	0.832

	Self-reported evoked pain (from the painDetect)			
	Cluster 1	Cluster 2	F	*P*
Temperature	2.1 ± 2.0 (0–5)	1.0 ± 1.3 (0–5)	15.76	**<0.001**
Light touch	1.2 ± 1.6 (0–5)	0.5 ± 1.2 (0–5)	9.64	**0.006**
Pressure	3.1 ± 1.4 (0–5)	2.2 ± 1.4 (0–5)	6.91	**0.017**

	Psychological factors				
	Cluster 1	Cluster 2	HC	F	*P*
HADS	12 ± 7 (0–26)*	10 ± 7 (0–24)†	5 ± 4 (0–21)*†	16.86	**<0.001**
PCS	18 ± 12 (1–42)*	14 ± 11 (0–38)†	5 ± 7 (0–23)*†	21.31	**<0.001**

Data are presented as mean (standard deviation, range). Significant results are printed bold.

* Post hoc analysis significance: HC-C1.

† Post hoc analysis significance: HC-C2.

HADS, hospital anxiety and depression scale; HC, healthy controls; PCS, pain catastrophizing scale; NRS, numeric rating scale.

In addition, Table 3 illustrates the differences in psychological factors between cluster 1, cluster 2, and HC. Both HADS and PCS were significantly different between the 3 groups (cluster 1, cluster 2, and HC). Post hoc analysis revealed that both patient clusters had higher HADS and PCS scores compared with HC (all *P* < 0.001), but there was no difference between the patient clusters themselves (HADS: *P* = 0.151; PCS: *P* = 0.206).

## 4. Discussion

This study aimed to investigate whether clusters of sensory phenotypes can be identified across 2 distinct chronic pain cohorts and whether these clusters relate to widespread hypersensitivities, pain characteristics, and psychological factors. Using cluster analysis, 2 different sensory phenotypes were identified across the 2 chronic pain cohorts. Interestingly, each cluster contained patients from both chronic pain cohorts (CRPS and LBP), implying common pathomechanisms across different chronic pain diagnoses. Cluster 1 presented with signs of central sensitization, evidenced by increased pain sensitivities in the control area, heightened maximal pain intensities, increased spatial pain extents, and more frequently reported evoked pain. Cluster 2 showed a sensory profile comparable to that of HC; thus, there was no identifiable sensory dysfunction based on QST.

### 4.1. From sensory phenotypes to potential mechanisms

Our findings suggest that in a chronic phase, even 2 distinct pain cohorts might share common underlying pathomechanisms. In such cases, the chronicity of the pathological condition with accompanying maladaptive reorganization of the nervous system could lead to a convergence of pathomechanisms. Moreover, one could argue that patients within the same cohort may experience chronic pain because of different pathomechanisms. The latter hypothesis supports the idea that chronic pain patients should be treated based on potential pathomechanisms inferred from sensory phenotypes and not only based on the clinical diagnosis.^15^ In doing so, the efficacy of pain treatment might increase.

The sensory phenotype of cluster 1 was similar to the “mechanical hyperalgesia” cluster reported by Baron et al.^3^ The “mechanical hyperalgesia” cluster showed increased mechanical pain sensitivity and reduced mechanical detection within the painful area. Mechanical hyperalgesia serves as the surrogate marker of central sensitization in human experimental pain models.^34,35^ Although mechanical hyperalgesia within painful areas could also be associated with peripheral sensitization, it is mainly considered a proxy for maladaptive changes in the central nervous system.^53^

The assumption of maladaptive central changes in cluster 1 is strengthened by the observed mechanical hypoesthesia. Previous literature argued that such hypoesthesia results from cortical reorganization because of continuous nociceptor activation and is referred to as pain-induced hypoesthesia.^11,22,35^ Moreover, animal experiments demonstrate the involvement of spinal mechanisms in mechanical hypoesthesia: C-fiber activation presynaptically inhibits nonnoxious transmission from peripheral afferent fibers to spinal projection neurons.^7,14,29^ Further evidence pointing towards central sensitization as pathomechanism of cluster 1 stems from the observed widespread hyperalgesia. This hypersensitivity in a remote pain-free area can be because of sensitized neurons in spinal (widespread spinal sensitization)^6^ or supraspinal regions^10^ or because of deficient endogenous descending inhibition.^40^ Our findings on signs of central sensitization, particularly widespread mechanical hyperalgesia, in patients with CRPS and LBP are well aligned with the previous literature, supporting central sensitization as a possible underlying pathomechanism in a subgroup of these patients (for review see: CRPS^5^; LBP^49^).

Interestingly, cluster 1 also displayed a higher maximal pain intensity and larger spatial pain extent compared with cluster 2. Prior research has postulated that elevated levels of clinical pain intensity may signify a condition of sensitized neurons within the central nervous system, given that pain intensity frequently fails to correspond with peripheral damage (eg, osteoarthritis).^2^ Regarding the spatial pain extent, previous studies demonstrated a relationship between maladaptive central processing and greater spatial pain extents in patients with neuropathic pain after spinal cord injury,^28^ pelvic pain,^32^ and fibromyalgia.^9^ Therefore, by further examining the pain characteristics between the clusters, these findings support the hypothesis that central sensitization could be an underlying pathomechanism in cluster 1. Here, it is important to consider that the relative proportion of patients with CRPS and LBP differed between the 2 clusters. Therefore, differences observed between the clusters (eg, spatial pain extent) may be attributed to the fact that cluster 1 comprised a relatively greater proportion of patients with CRPS than cluster 2, who generally exhibited larger spatial pain extents as demonstrated in Table 1. Although we have controlled for the chronic pain cohort as a confounding factor in our analysis, there could be some residual confounding.

Notably, cluster 1 also demonstrated heightened thermal pain sensitivity within the most painful area. Thermal hyperalgesia was previously hypothesized to be indicative of irritable nociceptors and hence peripheral sensitization.^26,42^ Thereby, both central and peripheral sensitization might contribute to the pathomechanisms of cluster 1. This assumption aligns with the observation of a “mixed phenotype” cluster, previously documented in a large cohort of individuals with CRPS.^12^

Cluster 2 displayed a sensory profile comparable to HC. Therefore, QST did not reveal any aberrant sensory function in approximately two thirds of the patient sample (51 of 78). Clusters of chronic pain patients with a normal sensory profile have previously been documented in patients with LBP,^38^ which represented more than 80% of cluster 2. Thus, it is possible that QST was not sufficiently sensitive to unmask pathomechanisms in cluster 2. Quantitative sensory testing primarily evaluate superficial afferent fiber function; hence, sensitization of deep afferent fibers might not be sufficiently uncovered. Alternatively, sensitization of the nociceptive neuraxis may not be the predominant factor causing pain in these patients. It is well documented that pain is a multidimensional phenomenon, including physiological/biological, psychological, and social factors (biopsychosocial model of pain).^21,36^ Therefore, the physiological aspects of pain processing might be considered normal in cluster 2, whereas other aspects, such as psychosocial factors, could contribute to the experience of chronic pain. In particular, psychological factors might play a pivotal role in the experience of pain regardless of the cluster allocation because psychological questionnaire scores were significantly higher in both clusters compared with HC. Therefore, effective treatment of chronic pain requires a comprehensive approach that addresses all factors of the biopsychosocial model.^30^

### 4.2. Sensory phenotypes on a single subject level

Participants with a significantly increased pain sensitivity in the control area were most frequently observed in cluster 1. This finding is well aligned with the abovementioned signs of central sensitization. Next to cluster 1, also approximately 20% of cluster 2 and HC demonstrated a gain of function in the control area, exceeding the expected 5% cutoff of 1.96SD. This gain of function could be attributed to deviations in the QST protocol, such as misalignment with the DFNS-based testing areas or protocol shortening. In addition, prior experimental testing (eg, neurophysiological assessments) might have influenced participants' sensitivity. To address these issues, we compared our patient data with that of HC, who underwent the same study procedure. Notably, however, the increased pain sensitivity observed in HC aligns with previous findings,^31^ which reported up to 30% of HC showing gain of function while following the regular QST protocol. Hence, the observed gain of function in HC might highlight a predisposition to develop chronic pain after an inciting event.^13^ For cluster 2, the gain of function observed in a pain-free control area additionally supports the possibility of central disinhibition, as discussed earlier.^16^

### 4.3. Limitations

There are some limitations to this study that should be acknowledged. First, the cluster analysis was conducted without a validation set. However, steps were taken to address this limitation. Specifically, the analysis was performed with bootstrapped data and a different clustering method (hierarchical), which helped ensure the robustness of the results. Second, no sample size estimation was performed before the study, potentially reducing the study's statistical power, and the cluster sample sizes were unequal, which could have introduced bias during comparison. To validate our findings, studies with larger sample sizes are needed. Finally, because patients were not taken off their pain medication owing to ethical concerns, medication may have influenced some of the study outcomes. Yet, we statistically accounted for this potential confounder and found no significant effect.

### 4.4. Conclusion

Using QST-based sensory phenotyping, shared pathomechanisms could be inferred across 2 distinct chronic pain cohorts. This finding highlights the need for future mechanism-targeted rather than diagnosis-dependent treatment approaches in chronic pain. In addition, many chronic pain patients presented with normal sensory phenotypes with no signs of sensitization within the somatosensory nervous system. Either QST was not sufficiently sensitive and more objective readouts are needed to reveal sensitization or it highlights that acute superficial sensory stimuli could be processed normally in these chronic pain patients. Hence, many chronic pain patients might not primarily have pain because of sensitized neurons within the nociceptive neuraxis but other factors, such as psychosocial ones, contribute to their pain.^30^

## Disclosures

The authors have no conflict of interest to declare.

## Appendix A. Supplemental digital content

Supplemental digital content associated with this article can be found online at http://links.lww.com/PR9/A211.

## Supplementary Material

SUPPLEMENTARY MATERIAL
