# Supplementary material for: Sensory phenotypes in complex regional pain syndrome and chronic low back pain—indication of common underlying pathomechanisms
Source: Pain Rep. 2023 Nov 15;8(6):e1110. doi: 10.1097/PR9.0000000000001110 (PMC10653599; doi:10.1097/PR9.0000000000001110)
Supplement: SUPPLEMENTARY MATERIAL [file painreports-8-e1110-s001.pdf]

# Supplementary information

*Table S1: Most painful and pain-free control area of chronic pain patients.*

| Most painful area | CRPS (N=19) | LBP (N=59) |
|---|---|---|
| Shoulder | 1 | - |
| Elbow | 1 | - |
| Forearm | 1 | - |
| Hand | 11 | - |
| Back L2 | - | 6 |
| Back L3 | - | 7 |
| Back L4 | - | 14 |
| Back L5 | - | 17 |
| Sacral | - | 8 |
| Buttocks | - | 7 |
| Foot | 5 | - |
| **Pain-free control area** | **CRPS (N=17)** | **LBP (N=59)** |
| Shoulder | 12 | - |
| Upper arm | - | 1 |
| Hand | 5 | 58 |

*Two CRPS patients did not have a suitable pain-free control area. One LBP patient had scar tissue on the hand and was tested on the upper arm as a control area. Abbreviations: CRPS: complex regional pain syndrome; LBP: low back pain*

*Table S2: Regular pain medication intake of chronic pain patients*

| Medication group | N |
|---|---|
| Anti-inflammatory and anti-rheumatic products | 11 |
| Analgesics | 11 |
| Opioidergic | 3 |
| Non-opioidergic | 5 |
| Both | 3 |
| Anticonvulsants | 10 |
| Psycholeptics | 5 |
| Psychoanaleptics | 15 |

*Table S3: Quality criteria provided by the NbClust() function.*

| Index | Optimal # of clusters | Index value | # cluster occurrence |
|---|---|---|---|
| Beale | 2 | -0.78 | 9 |
| CCC | 2 | 3.32 | |
| CH | 2 | 36.33 | |
| Duda | 2 | 1.51 | |
| Frey | 2 | 1.75 | |
| McClain | 2 | 0.57 | |
| PseudoT2 | 2 | -14.92 | |
| PtBiserial | 2 | 0.53 | |
| Silhouette | 2 | 0.3 | |
| Ball | 3 | 47.72 | 3 |
| Ratkowsky | 3 | 0.35 | |
| TrCovW | 3 | 821.32 | |
| Hartigan | 4 | 14.37 | 4 |
| KL | 4 | 14.76 | |
| Scott | 4 | 63 | |
| TraceW | 4 | 29.75 | |
| Marriot | 6 | 1.05E+07 | 3 |
| Rubin | 6 | -0.67 | |
| SDindex | 6 | 1.63 | |
| Dunn | 8 | 0.18 | 2 |
| Friedman | 8 | 3.95 | |
| Cindex | 10 | 0.35 | 3 |
| DB | 10 | 1.09 | |
| SDbw | 10 | 0.28 | |
| Dindex | NA | NA | |
| Hubert | NA | NA | |

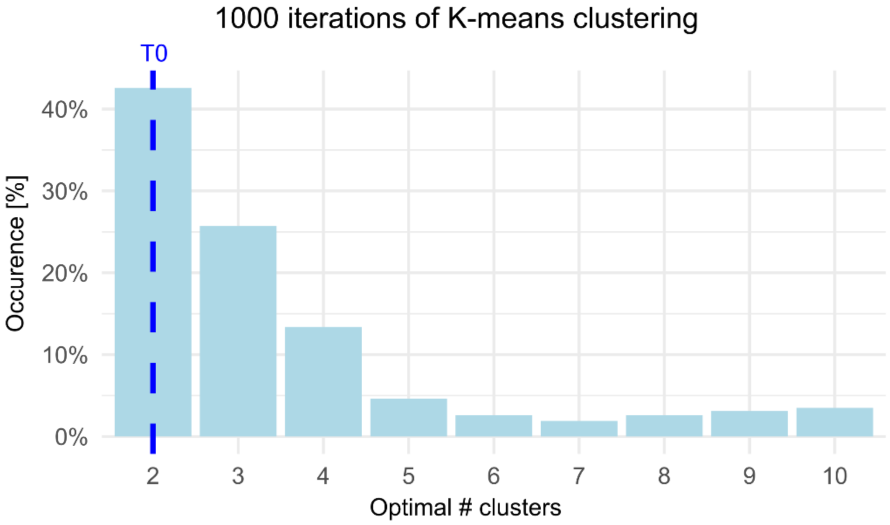


*Figure S1: 1000 iterations of K-means clustering based on bootstrapped data, T0 illustrates the optimal # of clusters based on the real data,*
